# Machine learning techniques for predicting neurodevelopmental impairments in premature infants: a systematic review

**DOI:** 10.3389/frai.2025.1481338

**Published:** 2025-01-20

**Authors:** Arantxa Ortega-Leon, Daniel Urda, Ignacio J. Turias, Simón P. Lubián-López, Isabel Benavente-Fernández

**Affiliations:** ^1^Intelligent Modelling of Systems Research Group, Department of Computer Science Engineering, Algeciras School of Engineering and Technology (ASET), University of Cádiz, Algeciras, Spain; ^2^Grupo de Inteligencia Computacional Aplicada (GICAP), Departamento de Digitalización, Escuela Politécnica Superior, Universidad de Burgos, Burgos, Spain; ^3^Biomedical Research and Innovation Institute of Cádiz (INiBICA) Research Unit, Puerta del Mar University Hospital, Cádiz, Spain; ^4^Department of Pediatrics, Neonatology Section, Puerta del Mar University Hospital, Cádiz, Spain; ^5^Paediatrics Area, Department of Mother and Child Health and Radiology, Medical School, University of Cádiz, Cádiz, Spain

**Keywords:** machine learning, preterm infants, neurodevelopmental impairment, NDIs prediction, NDIs prognosis

## Abstract

**Background and objective:**

Very preterm infants are highly susceptible to Neurodevelopmental Impairments (NDIs), including cognitive, motor, and language deficits. This paper presents a systematic review of the application of Machine Learning (ML) techniques to predict NDIs in premature infants.

**Methods:**

This review presents a comparative analysis of existing studies from January 2018 to December 2023, highlighting their strengths, limitations, and future research directions.

**Results:**

We identified 26 studies that fulfilled the inclusion criteria. In addition, we explore the potential of ML algorithms and discuss commonly used data sources, including clinical and neuroimaging data. Furthermore, the inclusion of omics data as a contemporary approach employed, in other diagnostic contexts is proposed.

**Conclusions:**

We identified limitations and emphasized the significance of employing multimodal data models and explored various alternatives to address the limitations identified in the reviewed studies. The insights derived from this review guide researchers and clinicians toward improving early identification and intervention strategies for NDIs in this vulnerable population.

## 1 Introduction

In this paper, we explore key topics related to preterm infants. We will review which factors are associated with long-term NeuroDevelopmental Impairments (NDIs) in this high-risk population and evaluate machine learning models applied to predict NDIs in very preterm infants (VPIs).

Very preterm infants are defined as those born before 32 weeks of gestation, and are exposed to a higher risk of NDIs. Early extrauterine life is associated with comorbidities and brain injury (George et al., 2018) which impact the long-term outcomes of very preterm infants (Franz et al., 2009). These patients require special care and interventions in the Neonatal Intensive Care Unit (NICU) to increase survival rates without NDIs. These interventions include respiratory and cardiovascular support and parenteral nutrition among others (Di Fiore et al., 2016). Patients are also exposed to painful procedures, such as venipuncture or heel stick for blood analysis (Ochiai et al., 2016), and are frequently handled for different procedures such as imaging (e.g., point-of-care ultrasonography, magnetic resonance imaging, ultrasound) (Hand et al., 2020), and hemodynamic and neurophysiologic monitoring (e.g. amplitude-integrated electroencephalography) (El-Dib et al., 2022).

Very preterm infants are at a higher risk of developing short- and long-term adverse neurodevelopmental outcomes, such as cognitive, motor, visual, and hearing impairments, compared with their full-term counterparts (Chung et al., 2020; Adams-Chapman et al., 2018). These NDIs are related to multiple antenatal, perinatal, and postnatal factors (Rogers and Hintz, 2016).

Several studies have considered clinical features as risk factors for NDIs, providing a better understanding of potential pathways to adverse outcomes in very preterm infants. Prenatal factors include maternal infections (Leviton et al., 2016), drug abuse (Manuck et al., 2014), hypertension (Nakamura et al., 2021), and malnutrition (Leviton et al., 2016; Vohr et al., 2017). Perinatal factors include lower gestational age (Salas et al., 2016; Larsen et al., 2022), sex (higher risk in males) (Agarwal et al., 2018), low five minute Apgar score (Khorram et al., 2022), high scores in scales such as the Clinical Risk Index for Babies (CRIB) score (Lodha et al., 2009). Comorbidities include necrotizing enterocolitis (Matei et al., 2020), early and late onset sepsis (Flannery et al., 2022), bronchopulmonary dysplasia (Ambalavanan et al., 2012), patent ductus arteriosus (Edstedt Bonamy et al., 2017), retinopathy of prematurity (Molloy et al., 2016) and brain injury (Rand et al., 2016; Glass et al., 2018). Moreover, other factors like long-term medical requirements and access to supportive therapies, which are associated with the patient's family socioeconomic status, might also influence NDIs (Rogers and Hintz, 2016; Benavente-Fernández et al., 2019, 2020).

Cranial Ultrasound (cUS) and Magnetic Resonance Imaging (MRI) help identify and monitor brain injury and have been widely investigated to identify early neuroimaging biomarkers that could be potential predictors for NDIs (Hintz et al., 2015; George et al., 2021; Parikh, 2016). Brain injury includes germinal matrix-intraventricular hemorrhage (Bolisetty et al., 2014), white matter injury (Schneider and Miller, 2019; Peyton et al., 2017; Kwon et al., 2014) with cUS being the most widely used tool because it is non-invasive, low cost, and available at the bedside. Brain injury detected via cUS is considered a significant predictor of NDIs in very preterm infants (Campbell et al., 2021; Wu et al., 2019; Law et al., 2021; Beunders et al., 2022). On the other hand, MRI is a neuroimaging technique that captures a detailed image of brain tissues and provides a better delineation of deep and cortical structures (Rogers and Hintz, 2016), which in turn makes MRI another valuable tool for NDIs risk prediction (Hintz et al., 2015; Schneider and Miller, 2019; Cayam-Rand et al., 2019; Kline et al., 2020).

Early and accurate diagnosis of NDIs in very preterm infants can provide a unique opportunity to improve short- and long-term clinical outcomes. Identifying the factors associated with later NDIs in VPIs can provide valuable knowledge to clinicians, leading to the design and application of neuroprotective strategies and early intervention. These neuroprotective therapies can benefit long-term neurodevelopmental outcomes (Jarjour, 2015; Crilly et al., 2021).

The prediction of later NDIs in very preterm infants is challenging. Clinicians usually use different scales, such as the Hammersmith Infant Neurological Examination (HINE) (Haataja et al., 1999), Neonatal Behavioral Assessment Scale (NBAS) (Brazelton and Nugent, 1995) and the General Movement Assessment (GMA) (Prechtl, 2001; Dubowitz et al., 1999), among others. In addition to clinical assessments, different methods have been studied and have demonstrated good potential for predicting NDIs in very preterm infants, such as early visuospatial attention (Beunders et al., 2021), and the visual tracking neonatal neurobehavior assessments (McGowan et al., 2022).

Artificial Intelligence (AI) is a rapidly evolving field that is transforming healthcare in many ways, including neonatal healthcare. The use of AI in the NICU can potentially improve patient outcomes, reduce treatment costs, and enhance the efficiency of care delivery. There are different aspects where AI is being implemented at NICUs including early diagnosis and outcome prediction (Son et al., 2022; Raimondi et al., 2018; Vats et al., 2022), monitoring (Lyra et al., 2022), neuromonitoring (O'Sullivan et al., 2023; Moghadam et al., 2021), and neuroimaging (Gruber et al., 2022; Shen et al., 2023). These studies are only examples of the potential of AI in neonatal healthcare, which is expected to lead to improvements in the diagnosis, treatment, and outcomes of preterm infants in a way that is both faster and more reliable. Machine Learning (ML), which is a branch of AI, has emerged as a powerful tool for predicting NDIs. By leveraging advanced algorithms and analyzing large-scale neuroimaging data, these techniques offer valuable insights into identifying early risk factors and accurately forecasting the likelihood of NDIs in individuals. These predictive models can assist healthcare professionals in making informed decisions, developing personalized interventions, and improving outcomes for individuals at high risk of NDIs (Bowe et al., 2023a; van Boven et al., 2022; Baker and Kandasamy, 2022).

Overall, although significant advances have been made in this field, there remains a need for consensus on strategies and methodologies (Baker and Kandasamy, 2022). One of the strategies currently applied is multimodal data integration (MDI). MDI is defined as the integration of various data types that, complement each other and provide significant information regarding a state, and MDI can potentially enhance the predictive power of ML models compared to a single data modality (Boehm et al., 2022).

Multimodal data integration involves the combination of heterogeneous information sources, such as electronic health records (e.g., prenatal and postnatal factors, sociodemographic data), neuroimaging data derived from MRI and ultrasound (e.g., morphometrics, volumetrics, structural and functional connectomes), electroencephalogram data (e.g., raw EGG traces) and omics data (e.g., DNA methylation, transcriptomic data, protein expression). Each of these modalities requires distinct preprocessing and feature extraction techniques. The subsequent merging of these information sources provides a holistic understanding of the individual's condition, enabling an accurate diagnosis, better clinical decision-making, and personalized treatment (Cui et al., 2023). Multimodal ML models can improve prediction accuracy by providing robust and reliable prediction models. By combining multiple data sources, multimodal models can identify complex patterns and relationships that may not be evident from a single data source. The integration of diverse data sources can potentially facilitate the identification of disease subtypes and enable the exploration of novel treatments for these conditions.

In this systematic review, we aimed to identify studies that employed ML algorithms to predict NDIs in very preterm infants and address the following key questions:

1. Which ML models have been utilized?
2. Which evaluation metrics were used to assess these ML models?
3. Do multimodal machine learning techniques exhibit superior performance compared to unimodal techniques in predicting neurodevelopmental impairments in very preterm infants?
4. What are the main limitations of these research studies?

This study aimed to comprehensively review the current state-of-the-art ML techniques for predicting NDIs in very preterm infants. We hypothesized that multimodal machine learning techniques will demonstrate superior predictive performance compared with unimodal techniques in predicting neurodevelopmental impairment in very preterm infants. We provide a summary of recent research in this area and a discussion of the challenges and limitations encountered. The remainder of this paper is structured as follows: Section 2 outlines the protocol used in the literature review and the selection criteria. Section 3, provides an in-depth analysis and comparison of the selected studies and their results. Section 4, discusses the advantages of multimodal data integration, presenting a critical discussion of the findings and analyzing challenges and potential future strategies in this field. Finally, in Section 5, we describe the conclusions of this review.

## 2 Materials and methods

### 2.1 Search criteria

This systematic review was conducted according to the Preferred Reporting Items for Systematic Review and Meta-Analysis (PRISMA) guidelines and was registered in PROSPERO on November 26th, 2023 (CRD42023483014). In alignment with the established best practices for systematic reviews, we conducted a comprehensive literature search using the following four databases: PubMed, Scopus, Web of Science, and IEEE Xplore. These databases were selected to ensure a wide range of relevant studies, thereby maximizing the best outcomes in this systematic review. The selected databases are well known across the scientific community and are described as follows.

- PubMed: A comprehensive database specializing in biomedical and life science literature, provided by U.S. National Institutes of Health, National Library of Medicine (NIH/NLM).
- Scopus: An extensive database encompassing interdisciplinary scientific research provided by Elsevier.
- Web of Science: Database scientific research covering a wide range of multidisciplinary fields provided by Clarivate.
- IEEE Xplore: A database focused on engineering and technology scientific research published by Institute of Electrical and Electronics Engineers (IEEE).

Our search criteria were developed through collaborative consensus, integrating clinical expertise, computer science insights, and the authors' domain knowledge to identify research articles addressing our questions effectively. Furthermore, in this review, we defined multimodal data models as those that incorporate multiple data types, such as clinical, neuroimaging, and omics data. The search criteria for each database are presented in Table 1.

**Table 1:** Search terms.

**Database**	**Query**
PubMed	((((“Infant, Premature” OR “Infant, Extremely Premature”) OR “Infant, Very Low Birth Weight”) OR “Cognitive Dysfunction”) OR “Psychomotor Disorders”) AND “Machine Learning”
Scopus	Infant, OR premature OR cognitive AND machine learning
Web of Science	Very Preterm OR Cognitive AND Machine Learning OR Very Preterm AND Machine Learning AND motor
IEEE Xplore	Very Preterm AND Machine Learning AND Cognitive OR Very Preterm AND Machine Learning AND motor

### 2.2 Selection criteria

The literature inclusion criteria (IC) were as follows:

- IC1: publication date between January 2018 and December 2023.
- IC2: research conducted in English.
- IC3: published in a peer-reviewed journal.
- IC4: focusing on multimodal or unimodal machine learning techniques for neurodevelopmental outcome prediction.
- IC5: addressing the prediction of neurodevelopmental impairment in very preterm infants (≤ 32 weeks) in longitudinal studies with follow-up until 18 months to 2 years corrected age.

**Figure 1:**
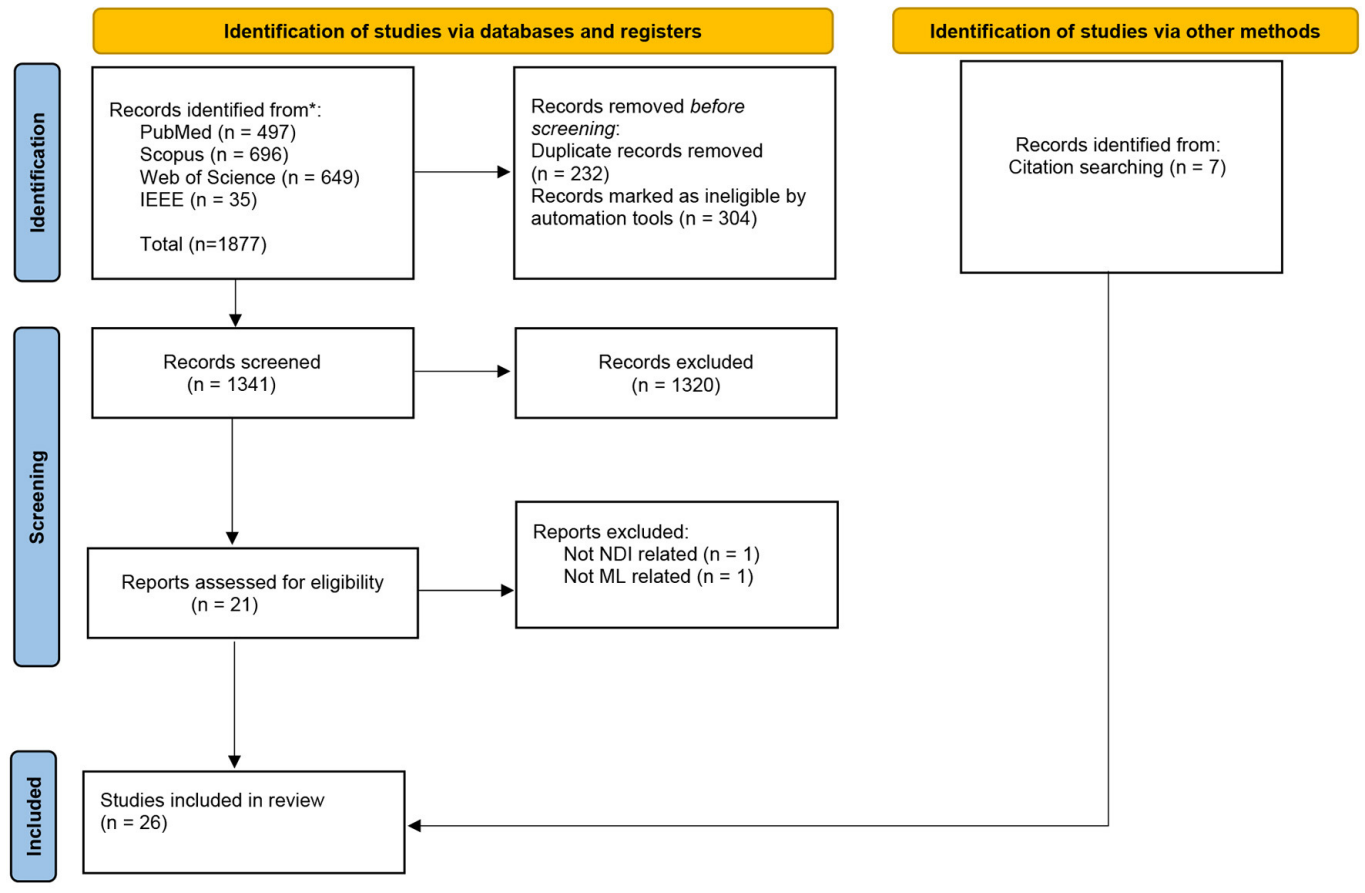
Flow diagram illustrating the literature selection process in accordance with PRISMA guidelines.

The flow diagram illustrating the literature selection is presented in Figure 1. Following the initial search, we identified a total of 1,877 research articles. Duplicate records were removed, and studies focused on cognitive decline in adults (e.g., Alzheimer's disease) were excluded using automated tools. After these exclusions, 1,341 articles remained. Two authors then independently screened these articles based on their abstracts, identifying 21 research studies that met the inclusion criteria. Additionally, seven more studies meeting the inclusion criteria were identified through alternative methods, such as citation searching.

## 3 Results

In this study, our primary objective was to evaluate whether multimodal ML techniques perform better than unimodal ML techniques in predicting NDIs in very preterm infants. Furthermore, we specifically focused on the key limitations of the included studies. We categorized three primary drawbacks: (1) the absence of integrated multimodal data in the models; (2) the persistent challenge of limited sample sizes, a common constraint in clinical research; and (3) the oversight in integrating prior knowledge, including aspects such as feature selection or the utilization of pre-trained models.

**Figure 2:**
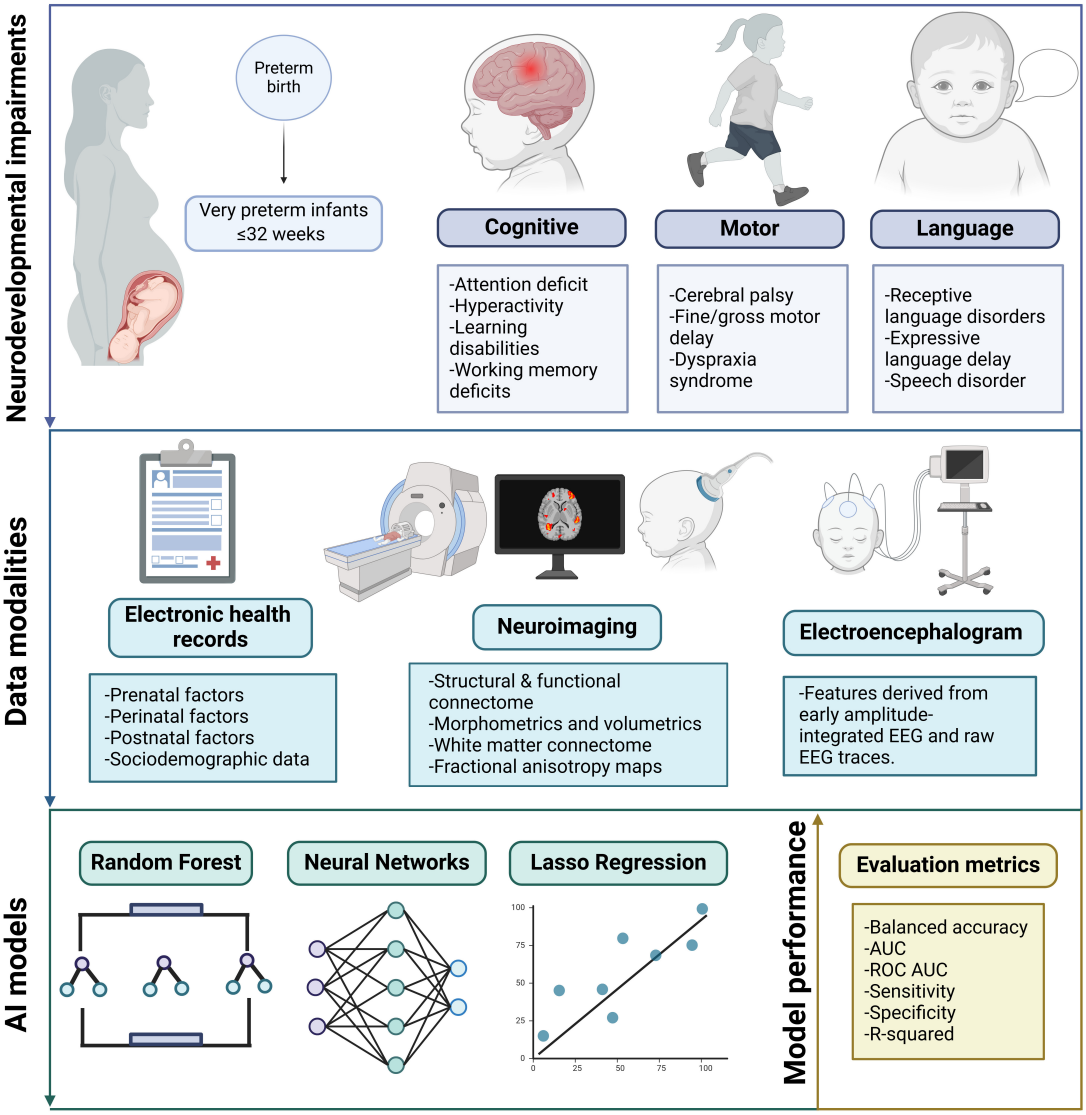
Overview of predictive models for neurodevelopmental impairments in very preterm infants. This figure summarizes findings from a systematic review, highlighting the types of neurodevelopmental impairments, data modalities used, and common artificial intelligence models employed in predicting outcomes for very preterm infants. Created in BioRender. Ortega, A. (2024) https://BioRender.com/o15o656.

Most of the studies included in this review focused on very preterm infants (≤ 32 weeks of gestational age) and extremely preterm infants (≤ 28 weeks of gestational age). Moreover, neurodevelopmental assessments were performed, such as the Bayley Scales of Infant and Toddler Development-Third Edition (Bayley-III) (Bayley, 2006), Denver Developmental Screening Test II (Frankenburg et al., 1992), Mullen Scales of Early Learning (Mullen et al., 1995) and Kyoto Scale of Psychological Development (Ikuzawa et al., 2002). In most of the studies, these neurodevelopmental assessments were performed at 2 years corrected age, whereas in one study, they were conducted at 36 months. The characteristics from the included articles were extracted and synthesized in Table 2. This table cover a range of attributes, such as the data modalities used, type of NDIs, methods, results, cohort size, and drawbacks. Figure 2 summarizes predictive models for neurodevelopmental impairments in very preterm infants, highlighting the type of NDI, data modalities used, and AI models.

**Table 2:** Literature review.

**Ref**.	**Data modalities**	**NDIs**	**Methods**	**Results**	**Cohort size**	**Main drawbacks**		
						**Multi-modal**	**Sample size**	**Prior knowledge**
Ali et al. (2022)	Brain functional connectome	Cognitive	Self-training Deep Neural Network	Acc:71.0%, Sen:70.4%, Spe:71.5%, AUC:0.75	103	x	x	x
Bowe et al. (2023b)	Clinical and sociodemographic data	Cognitive	Logistic Regression, Random Forest, Support Vector Machine, Gradient Boosting	AUROC: 0.77, Sen:0.93, Spe:0.46,	1062	x		
Brown et al. (2019)	Brain structural connectome	Cognitive and motor	Lasso regression	Motor r=0.44, AOC:14.00, Acc:72.50 Cognitive r=0.44, AOC:15.36, Acc:59.50	168	x	x	
Chen et al. (2020)	Brain structural connectome	Cognitive	Transfer Learning Enhanced Convolutional Neural Network	Bal.Acc:74.5%, Spe:78.7%, Sen:70.2%, AUC:0.75	110	x	x	x
Chen et al. (2022)	Brain structural connectome	Cognitive	Connectome-Inception Deep Convolutional Neural Network	Acc:81.6%, Spe:83.6%, Sen:78.3%, AUC:0.81	80	x	x	x
Demirci et al. (2023)	Perinatal and longitudinal data	Mental and psychomotor	Random Forest classifier	Bal.Acc mental: 72%, Spe:54.8%, Sen:88.7%, Bal.Acc psychomotor: 73%, Spe:51.7%, Sen:93.9%,	1109	x		x
Girault et al. (2019)	White matter connectome	Cognitive	Dense neural network	Acc 83.8%	37	x	x	
He et al. (2018)	Brain functional connectome data	Cognitive	Stacked Sparse Autoencoder based Artificial Neural Network framework	Acc:70.6%, AUC:0.76, Sen:70.1%, Spe:71.2%	28	x	x	
He et al. (2020)	Clinical, demographic data and brain connectome	Cognitive, motor and language	Deep transfer learning neural network	Cognitive AUC:0.86, Language AUC:0.66, Motor AUC:0.84	291		x	
He et al. (2021)	Structural and functional brain connectome, clinical data	Cognitive, motor and language	Deep multimodal learning model	Cognitive Acc:88.4%; Language Acc:87.2%; Motor Acc:86.7%	261		x	x
Janjic et al. (2020)	MR spectroscopy and DTI	Cognitive and motor	Single-hidden-layer feedforward neural networks (fNNs)	Sen:100%, Spe:100%, PPV:100%, NPV:99.1%	127	x	x	x
Juul et al. (2023)	Clinical and demographic data	Cognitive, motor and language	Bayesian Additive Regression Trees	AUROC:0.87, Sen:84.6%, Spe:72.3%	692	x		
Kline et al. (2020)	Brain morphometric, volumetrics biomarkers from MRI and clinical features	Motor	Lasso regression and multivariable linear regression	Regional cortical surface area p=0.30 to 0.57; subcortical volumes p=0.29 to 0.55; cortical curvature -0.26 to -0.43.	75		x	
Li et al. (2022)	Quantitative brain maturation and geometric features from structural MRI	Cognitive	Ontology-guided Attribute Partitioning-Ensemble Learning	Acc:71.3%, Sen:70.6%, Spe:72.6%, AUC:0.74	110	x	x	
Li et al. (2023)	Brain structural connectomes	Motor	Semisupervised graph convolutional network	Acc:68.0%, Bal.Acc:66.7%, Sen:63.1%, Spe:70.2%, AUC:0.69	224	x	x	x
Ouyang et al. (2020)	Regional cortical microstructure markers from diffusion MRI	Cognitive, motor and language	Support vector regression (SVR) and leave-one-out cross-validation	Cognitive-r=0.53; Language-r=0.47; Motor-r=0.1,	46	x	x	x
Pagnozzi et al. (2023)	Brain morphometrics and clinical data	Cognitive, motor and language	Lasso regression	Motor r=0.51; Cognitive r=0.48; Language r=0.36;	181		x	
Raghuram et al. (2022)	Mean velocity in the vertical direction, median, standard deviation, and minimum quantity of motion	Cerebral palsy	Multivariable regression	Sen:55%, Spe:80%, PPV:26%, NPV:93%, C-statistic:0.74	252	x	x	x
Routier et al. (2023)	Brain function and structure information, perinatal and postnatal risk factors	No or moderate NDIs, death or severe NDIs.	Classification and regression tree	Acc:87.2%, Spe:88.0%, Sen:86.4%, AUC:91.7, NPV:85.5, PPV:89.5	109		x	
Saha et al. (2020)	Fractional anisotropy maps derived from diffusion MRI	Motor	Convolutional Neural Network (CNN)	Mean Sen:70%, Mean Spe:74%, Mean AUC:72%, F-score:68%, Mean Acc:73%	77	x	x	x
Schadl et al. (2018)	White matter microstructure derived from DTI	Cognitive and motor	Logistic regression with feature selection and leave-one-out cross-validation	Cognitive-Sen:100%, Spe:100%, AUC:1; Motor-Sen:90%, Spe:86%, AUC:0.91	60	x	x	x
Ushida et al. (2023)	Clinical data	Cognitive, cerebral palsy	Six ML models including gradient boosting decision tree (GBDT)	GBDT AUROC: 0.750	13,751	x		x
Valavani et al. (2021)	Clinical, demographic data and diffusion MRI features	Language	Boruta, ReliefF expRank, Random Forest variable importance	Bal.Acc:91%, Sen:86%, Spe:96%,	89		x	
Vassar et al. (2020)	Structural MRI and white matter microstructure from DTI	Language	Multivariate logistic regression with feature selection and leave-one-out cross-validation	Sen:89%, Spe:86%, AUC:0.916	102	x	x	x
Wagner et al. (2022)	Clinical data and MRI-based radiomics	Cognitive, motor and language	Elastic Net	Cognitive-AUROC:0.79; Motor-AUROC:0.83; Language-AUROC:0.64	166		x	x
Wang et al. (2023)	Early amplitude-integrated EEG & raw EEG	Cognitive and motor	Support vector regression model, histogram-based gradient boosting classification model	Bal.Acc:0.77 to 0.81	369	x	x	x

AUC: area under the curve, AOC: area over the curve, AUROC: area under the receiver operating curve, Acc: accuracy, Bal. Acc: balanced accuracy, EEG: electroencephalogram, Spe: specificity, Sen: sensitivity, PPV: positive predictive value, NPV: negative predictive value.

### 3.1 Multimodal vs. unimodal models for predicting NDIs in very preterm infants

Research on very preterm infants for predicting NDIs using heterogeneous data and implementing Machine Learning (ML) and Deep Learning (DL) methods has mainly focused on integrating clinical data and MRI-based data. Some studies that have used multimodal data integration have utilized DL architectures (LeCun et al., 2015). Such is the case of the study conducted by He et al. (2020), a deep neural network and deep transfer learning neural network techniques were employed, utilizing clinical, demographic, and brain connectome data to predict cognitive, motor, and language impairments. Their model took advantage of both supervised and unsupervised learning for annotated and non-annotated data during model training by incorporating both supervised and unsupervised learning. The initial deep neural network prototype was pre-trained using supervised learning on 884 older children and adults diagnosed with autism. Later, the model was re-trained in an unsupervised manner using 291 VPIs, fine-tuned, and validated with supervised learning on 33 VPIs. Their findings demonstrated that the multimodal data model exhibited superior performance compared with their counterpart unimodal models: connectome features and clinical feature-based models.

Moreover, a study by He et al. (2018) proposed an Artificial Neural Network (ANN) (Rumelhart et al., 1986), to predict cognitive impairment in VPIs. They perform feature selection using a Stacked Sparse Autoencoder (SSAE) and outcome prediction using a Support Vector Machine (SVM) (Cortes and Vapnik, 1995). The framework included an unsupervised SSAE model that utilized functional connectome data from 884 participants in the autism brain imaging data exchange database. The SVM classifier was employed for cross-validation using a group of 28 very preterm infants to predict cognitive deficits. They found that using 90 regions of interest in the brain functional connectome data, they were able to obtain notable accuracy. In addition, the multimodal model outperformed other baseline models: clinical features, raw functional connectome features, and PCA with top components (Jolliffe, 2002). Furthermore, He et al. (2021), employed a deep multimodal learning model that uses structural and functional brain connectome data alongside clinical features to predict cognitive, motor, and language impairments. The model comprised a feature extractor and a fusion classifier, which efficiently discriminated across high-dimensional data types. This study also highlighted the advantages of the multimodal data model over the unimodal models: functional connectome, structural connectome, and clinical feature-based model.

The study of Pagnozzi et al. (2023) proposed a method that aimed to identify early MRI biomarkers. For their model, they included advanced structural MRI pre-processing steps to standardize the data and the state-of-the-art human connectome pipeline. The authors used the Least Absolute Shrinkage and Selection Operator (LASSO) model to predict cognitive, motor, and language impairments. Moreover, they included covariates (clinical features) in their models to compare different combinations of MRI features and covariates. Their findings suggest that the model that incorporates both data types outperforms the model that is based only on covariates.

Moreover, Kline et al. (2020), conducted a research study with the objective of developing a pipeline that extracts brain volumetrics and cortical morphometrics from structural MRI data. The authors applied the LASSO model to identify the most effective predictive biomarkers for motor development in VPIs. The researchers discovered that certain brain structures, such as the regional cortical surface area, displayed a positive association with motor development, whereas subcortical volumes exhibited a negative association. Additionally, the authors utilized multivariate regression analysis to identify specific structures, including thalamic volume, temporal lobe curvature, insula curvature, as independent predictors biomarkers of motor development. They found that models with both morphometrics and key covariates (clinical features) obtained better results than models with only morphometrics.

Routier et al. (2023) conducted a study to apply a multimodal model that combined unsupervised multivariate, classification, and regression tree analyses (CART). The study used brain structure and function data derived from cUS and electroencephalography (EEG), and perinatal and postnatal risk factors. Their aim was to predict two distinct outcome categories: favorable outcomes (no or moderate NDIs) and adverse outcomes (death or severe NDIs) in extremely preterm infants. They compared the different unimodal models against the multimodal model. Their findings showed that the multimodal model led to a significant increase in prediction accuracy compared with unimodal models: perinatal, postnatal, brain structure, and brain function-based models.

Furthermore, Wagner et al. (2022) implemented an Elastic Net model and leave-one-out cross-validation that used radiomics features derived from MRI and clinical features to predict motor, cognitive, and language outcomes in VPIs at 18 months and 33 months, and 4.5 years. The proposed multimodal model was compared with unimodal models, and the results indicated that using both features outperformed the unimodal models: MRI-based radiomics, gestational age, and a clinical features-based model. The research by Valavani et al. (2021) aimed to predict language deficiency outcomes based on multimodal data, including diffusion tensor imaging (DTI), clinical, and demographic features. They compared different selection algorithms including Random Forest, Boruta, and ReliefF expRank. Moreover, they identified eight clinical characteristics and imaging biomarkers that best predicted language impairment. Their results indicated that the multimodal model obtained a higher balanced accuracy, which outperformed the unimodal models.

### 3.2 Sample size

A common limitation encountered among the selected studies was the sample size, which is a well-known disadvantage of training machine learning models in clinical studies (Riley and Collins, 2023). The included studies in this review, only two had a considerable size cohort (Ushida et al., 2023; Bowe et al., 2023b; Demirci et al., 2023; Juul et al., 2023, while the rest of the studies had a population below of n=300. The longitudinal design of studies, examining the short and long-term effects of preterm birth offers invaluable insights into the trajectory of health outcomes over their life course. However, such studies are inherently challenged by attrition, where participants may drop out over time, thereby compromising the cohort size.

Attrition rates are influenced by various factors, including socioeconomic status and maternal education level. Additionally, it was observed that both healthy and unhealthy participants were likely to drop out (Teixeira et al., 2021). These losses to follow-up not only skew the representativeness of the sample but also introduce biases into the model's predictions, compromising the validity of the findings.

### 3.3 Prior knowledge

Healthcare data are inherently complex, and they involve numerous parameters that often result in a large number of features but with a small sample size. As dimensionality increases, the likelihood of individuals carrying specific combinations of these features decreases, creating blind spots–contiguous regions of feature space without any observations (Acosta et al., 2022; Berisha et al., 2021). This phenomenon is known as the “curse of dimensionality” (Bellman and Kalaba, 1959).

Various techniques are available to address this challenge, such as feature extraction and feature selection techniques. Furthermore, different approaches to dimensionality reduction in multimodal healthcare data have been reviewed by Acosta et al. (2022), highlighting methods that can learn abstract representations of clinical and biological data.

Using domain knowledge for feature engineering and selection ensures that the most relevant features are emphasized, which enhances the model's predictive power (Acosta et al., 2022). Feature selection in the context of predicting NDIs in very preterm infants has been used in clinical and sociodemographic data, demonstrating its usefulness for building effective predictive models. The study by Bowe et al. (2023b) aimed to construct a prediction model with various 90 variables, including sociodemographic and clinical information related to pregnancy, delivery, and neonatal care. These variables were selected based on the contents of the Swedish Neonatal Quality Register, existing literature, plausible hypotheses, and expert input. Moreover, Routier et al. (2023), highlighted the use of variable selection for perinatal and postnatal risk factors as unimodal prognostic models, as well as the research by Kline et al. (2020) that used key clinical features (male sex, gestational age, and global injury score on structural MRI) in their prediction model.

In addition, Juul et al. (2023) demonstrated feature selection by comparing models with different sets of features. They compare a 5-clinical feature model, 21 pre-selected clinical features, and hypothesis-free variable selection using a Bayesian Additive Regression Trees (BART) model. However, the model that obtained better results was the hypothesis-free variable selection. Furthermore, Valavani et al. (2021), which constructed a model with DTI-derived and clinical and demographic features and performed feature selection. This selection was based on existing literature that correlates biological and environmental factors with neurocognitive outcomes in very preterm infants.

In the neuroimaging context, the research by Kline et al. (2020) predicted NDIs based on brain volumetrics and cortical morphometrics derived from structural MRI. They applied prior knowledge on the selection of cortical morphometrics by selecting four features known to be altered in prematurity: surface area, gyrification index, sulcal depth, and inner cortical curvature. Moreover, Pagnozzi et al. (2023) approach which integrated early brain morphometrics derived from structural MRI with key clinical covariates, which were highlighted in their study as important drivers of the results. Furthermore, Brown et al. (2019) identified anatomical subnetworks of the human connectome derived from diffusion magnetic resonance imaging. Their model is based on previous work and introduces novel connectivity priors. The conducted experiments validate the hypothesis that incorporating prior knowledge improves the prediction accuracy.

Pretrained models leverage prior knowledge by typically being trained on large, accessible datasets. This is particularly advantageous in fields like clinical studies, where data scarcity can impede the training of ML models due to limited sample sizes. For instance, Chen et al. (2020), developed a model comprising two modules: a pre-trained deep convolutional neural network (CNN), which utilizes supervised learning on 1.2 million images from the ImageNet database, and a shallow CNN, which underwent training and refinement using VPIs brain connectome data.

Furthermore, Girault et al. (2019) developed their model based on white matter connectome derived from diffusion MRI. The model was first trained with full-term born infants and then applied to preterm infants. In their study, He et al. (2018) trained the model using unsupervised learning on brain connectome data from 884 subjects in the Autism Brain Imaging Data Exchange (ABIDE) database. Subsequently, cross-validation was performed on 28 very preterm infants. Additionally, He et al. (2020) incorporated supervised pre-training on 884 children and adults diagnosed with autism, which were also sourced from the ABIDE database. Subsequently, the model was re-trained unsupervised using data from very premature infants.

The study by Li et al. (2022) presents the Ontology-guided Attribute Partitioning (OAP) method, which is intended to improve feature subset delineation. This method was used to develop an ensemble learning framework for predicting cognitive NDIs using quantitative structural magnetic resonance imaging. Their methodology incorporates prior-defined ontologies describing brain parcellation and geometry/maturation. Their findings underscore the effectiveness of ontologies in terms of enhancing predictive modeling compared to traditional ML models.

## 4 Discussion

This study highlights the diverse machine learning and deep learning models employed to predict neurodevelopmental outcomes in very preterm infants. These include conventional methods like Random Forest (RF), Lasso, Elastic Net, Support Vector Regression (SVR) and logistic regression, alongside advanced techniques such as convolutional neural networks (CNN), deep neural networks (DNN) and feedforward neural networks (fNNs) among others. These models are particularly suited for analyzing neuroimaging data, the most common type of data modality alongside clinical data. Many studies demonstrate their potential, offering enhanced predictive accuracy and the ability to integrate complementary data sources. It is important to note that various metrics are used to evaluate the effectiveness of ML/DL models in this domain, depending on whether the objective involves classification or regression into predicting NDI scores. Metrics are chosen based on the specific task, with common measures including accuracy, balanced accuracy, sensitivity, specificity, F1 score, R-squared, AUC, and ROC AUC, each offering insights into model performance.

The hypothesis that multimodal models outperform unimodal models is supported by several studies, which demonstrate improved performance when multiple data modalities are used compared to single-modality approaches, as discussed in Section 3.1. However, achieving broad consensus on this finding remains challenging due to the limited availability of public datasets and the variability in testing conditions, which complicates direct comparisons across studies. Despite these efforts, current multimodal ML models for predicting neurodevelopmental impairments in very preterm infants remain relatively limited. Nonetheless, research indicates that multimodal data models generally outperform unimodal models, reinforcing the significance of integrating multiple data types for more accurate predictions into diagnosing NDIs.

Even with the considerable results obtained by the included studies featured in Section 3, these studies share several drawbacks, such as a small sample size, the absence of prior knowledge, and the lack of external validation. In this context, it is crucial to evaluate the robustness and effectiveness of these models through external validation on larger cohorts.

Developing machine learning models for predicting NDIs still faces various challenges that need to be addressed to improve the models and enable their implementation in healthcare institutions. Second, beyond data acquisition, another challenge is the imbalance in datasets. Due to the scarcity of patients with specific diseases or conditions within a particular cohort, the diseased population is significantly smaller than the healthy population. ML models learning from imbalanced datasets may be biased toward the majority class, leading to inaccurate predictions and sub-optimal performance for the minority class. Data augmentation techniques are highly valued under these conditions and have been already implemented before (Xiao et al., 2018; Chlap et al., 2021).

Transfer learning (TL) has also emerged as an approach for tackling imbalanced datasets. TL involves using pre-trained models or learned representations to enhance the performance of a new task with limited data. One of the advantages of TL is that it enables the transfer of knowledge from one healthcare institution to another, especially in cases where data sharing is limited due to privacy concerns. By training models on larger and more diverse datasets across different institutions and fine-tuning them on local data, healthcare organizations can benefit from the collective knowledge and experiences captured in the models without the necessity of sharing data.

Foundation models are ML models trained on large data, typically using self-supervised learning techniques, and can be adapted to different tasks. One remarkable feature of foundation models is their adaptability. Foundation models can learn from a diverse range of data types, such as text, images, and audio; when these models are fine-tuned on specific datasets, they can apply their generalized knowledge to specific domains.

These foundation models have proven highly effective in generating notable models with applications in healthcare (Bommasani et al., 2021). Significant advances in applications have been observed in the analysis fMRI (Caro et al., 2024), electronic medical records (Guo et al., 2023) and omics data (Dalla-Torre et al., 2023). Moreover, healthcare data are inherently multimodal, encompassing various types of data per individual. Thus, multimodal foundation models present a promising approach to enhance outcomes in this field (Bommasani et al., 2021; Lu et al., 2022).

The application of foundation models in healthcare has notably demonstrated their versatility; however, their potential in neonatal healthcare remains unexplored. Future studies in this area could pave the way for significant advancements by developing predictive models tailored to neonatal healthcare. These models could help in early diagnosis, potentially improving outcomes in this field.

Moreover, one notable limitation observed in the reviewed studies was the absence of external validation. External validation is a crucial step in model development because it assesses the generalizability of the model beyond the original dataset and determines its performance in different settings. It is essential to test the model's effectiveness in additional sites, considering the variations in equipment and assessments that may exist across different datasets. Without external validation, it is challenging to determine the model's reliability and applicability in real-world scenarios. Replicating studies remains a challenge, as demonstrated by Gondová et al. (2023), who were unable to reproduce the results of the reference study conducted by Ouyang et al. (2020), highlighting the difficulties in implementing and validating these models.

One approach that can be applied to external validation procedures is federated learning. Federated learning (FL) is a decentralized ML approach that allows multiple parties to collaboratively train a shared model without sharing their data, thereby ensuring data privacy and reducing the need for data transmission to a central server. This approach has been successfully applied in various healthcare studies (Antunes et al., 2022; Pfitzner et al., 2021; Rieke et al., 2020).

In addition, the use of synthetic patient data can significantly impact the pace of research and model development (Goncalves et al., 2020). Synthetic data have been proposed as a way to augment limited real data because it is difficult to complement scarce real data when obtaining datasets (Perez and Wang, 2017). Additionally, there have been explorations into using synthetic data for transfer learning from synthetic to real data, to improve ML algorithms in healthcare. Models such as Multimodal Neural Ordinary Differential Equations (MultiNODEs) (Wendland et al., 2022), Generative Adversarial Networks (GAN) (Yang et al., 2019), and diffusion models (Kotelnikov et al., 2023) have demonstrated successful application of these methods.

Regardless of the different techniques and frameworks available to overcome such challenges, future research should also focus on regulation and collaboration among healthcare institutions. Increasing the sample size in population studies will become feasible with enhanced support for data collection, long-term patient follow-up studies, and integration of regional and national collaborative initiatives for sharing data and collectively validating models to ensure their efficacy across diverse cohorts (Ngiam and Khor, 2019; Agrawal and Prabakaran, 2020). Additionally, establishing standardized protocols and data-sharing agreements can streamline research, improve data quality, and encourage innovation. By overcoming these obstacles, collaborative efforts are needed to advance the field by driving progress toward improving healthcare, especially in high-risk populations such as very preterm infants.

### 4.1 ML models for predicting NDIs using multimodal data, beyond neuroimaging and clinical features

**Figure 3:**
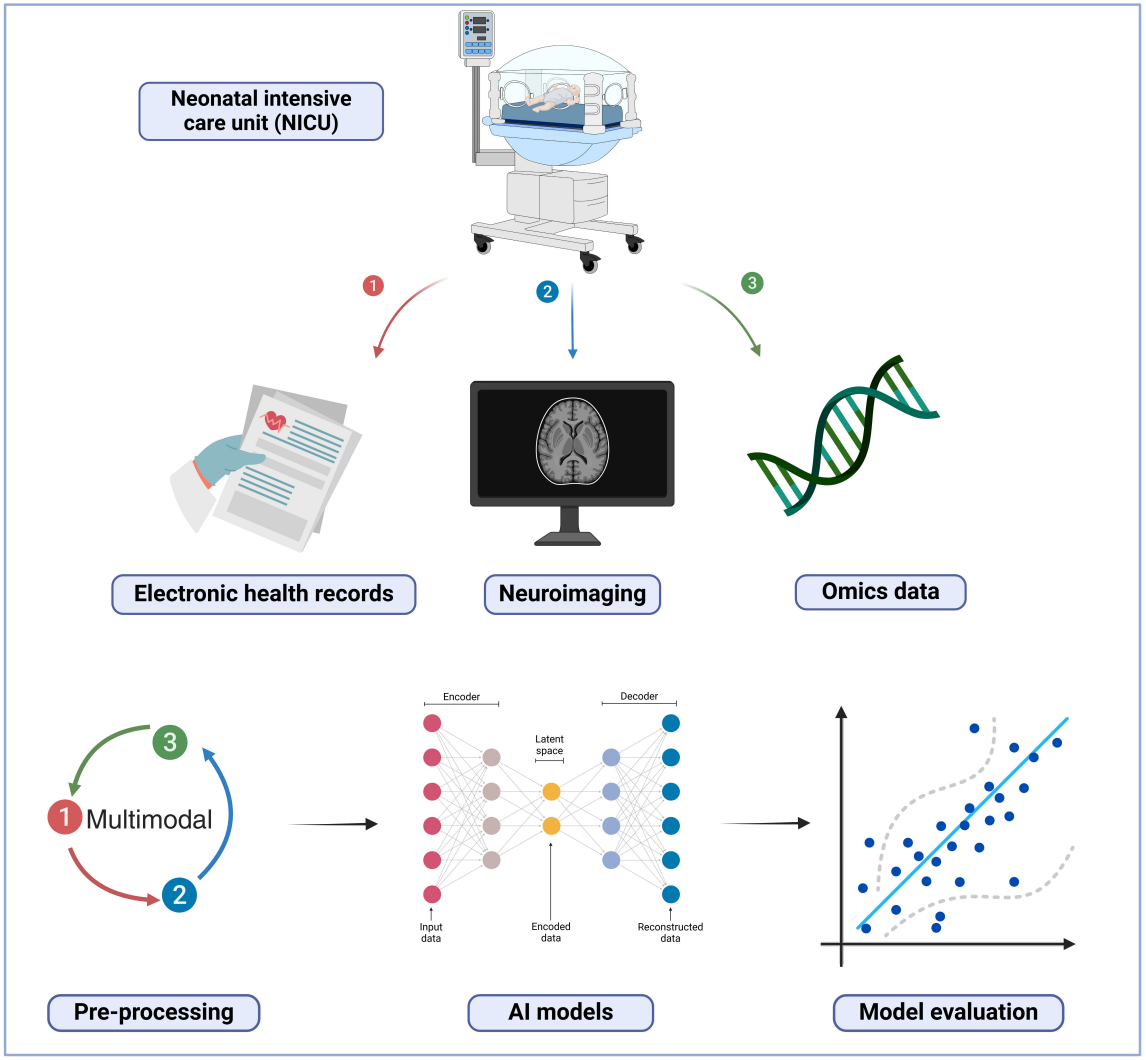
Multimodal machine learning model. Created in BioRender. Ortega, A. (2025) https://BioRender.com/t71k910.

A common factor identified across the included studies was the absence of omics data for predicting NDIs in very preterm infants. Analyzing omics data collections can be highly valuable for identifying previously understudied significant variables that may have a significant impact on NDIs (Khodosevich and Sellgren, 2023), as displayed in Figure 3. Multimodal data integration with the inclusion of omics features provides complementary information about a given state, leading to precise data-driven predictions (Li et al., 2016). This approach has been successfully employed in the study of diverse and complex diseases, such as cancer (Lipkova et al., 2022), Alzheimer's disease (Venugopalan et al., 2021), and neuropsychiatric disorders (Ghosal et al., 2021). Moreover, the authors concluded that their ML models combining these different layers of data were more accurate in terms of prediction than those based only on clinical variables, as it enable a comprehensive understanding of complex systems from multiple perspectives and levels.

#### 4.1.1 Omics biomarkers associated with NDIs in preterm infants

Potential genetic biomarkers of the risk of neurodevelopmental outcomes in preterm infants have also been considered in previous studies, such as the cases of the MET, NRG3, and SLC6A4 genes. These genes have been studied and found to be consistently associated with neurodevelopmental outcomes. This can lead to a risk profile of preterm infants (Blair et al., 2016). Furthermore, IL6R gene polymorphism has been characterized for psychomotor delay in preterm infants (Clark et al., 2018). FKBP5 gene polymorphism has also been characterized to contribute to the effects of early procedural repetitive stress on neurodevelopment in very preterm infants (D'Agata et al., 2017). Epigenetic mechanisms have also gained significance in NDIs research in preterm infants. DNA methylation plays an important role in brain development; thus, these epigenetic patterns may decode the basis of fetal brain development (Spiers et al., 2015).

Research studies have identified potential epigenomic predictors of cerebral palsy in newborns. A study indicated methylation of CSRP1 and USP44 genes in patients diagnosed with cerebral palsy born preterm compared with those born preterm without cerebral palsy (Massaro et al., 2021). Moreover, one study identified that microRNAs (miRNAs) such as miR-1469 and non-coding RNAs (ncRNAs) like NCRNA00171 and NCRNA00028 were significantly associated with cerebral palsy in newborn patients in their study cohort (Bahado-Singh et al., 2019). Additionally, it has been postulated that epigenetic modifications, such as the methylation of SLC6A3, can influence the neurodevelopment of impairments in preterm infants. In the study of Arpón et al. (2018) methylation levels of SLC6A3 were evaluated in relation to BSID-III in preterm patients at the age of 24-36 months. Another study identified 10 gene bodies and promoters of protein-coding genes related to neural development and function in preterm infants (Sparrow et al., 2016). In addition, another study conducted an epigenome-wide association study (EWAS) in preterm infants and identified target genes related to neurodevelopmental disorders (Wheater et al., 2022).

## 5 Conclusions

Machine Learning techniques have shown promising results in predicting NDIs in very preterm infants. In particular, multimodal data models have demonstrated significant results over unimodal data models. However, there are still challenges that need to be addressed to improve the models and implement them effectively in healthcare institutions. The reviewed studies lack external validation, which is crucial for assessing the generalizability of the ML models beyond the original dataset. External validation tests the model's effectiveness in different settings, considering variations in equipment and assessments across datasets.

Addressing the challenges of imbalanced datasets, external validation, and privacy concerns, along with exploring approaches like transfer learning and synthetic data, can significantly improve ML models for predicting NDIs in very preterm infants and enhance healthcare outcomes. Moreover, collaborative efforts among healthcare networks can help address challenges related to thorough, continuous, and accurate data analysis. By combining resources, sharing data, and collectively validating models, healthcare networks can drive progress, especially in critical patient populations like very preterm infants.
